# Supplementary material for: Improving HIV outcomes in Miami’s Black populations with clinic-based community health workers protocol: The integrated navigation and support for treatment adherence, counseling, and research (INSTACARE) randomized controlled trial
Source: PLoS One. 2025 Apr 7;20(4):e0316300. doi: 10.1371/journal.pone.0316300 (PMC11975065; doi:10.1371/journal.pone.0316300)
Supplement: S1 File — (DOCX) [file pone.0316300.s001.docx]

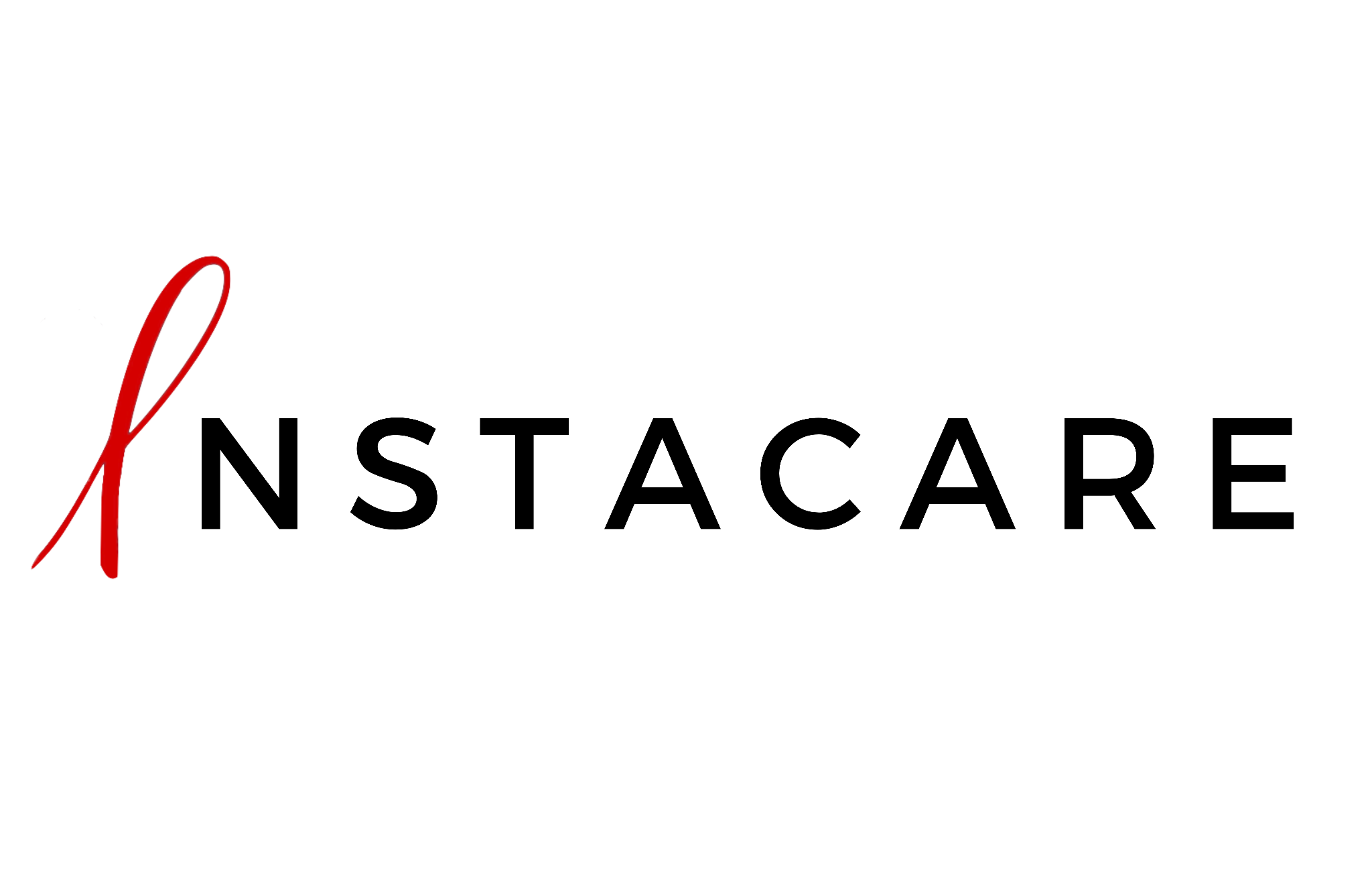


**Integrated Navigation Services for Treatment Adherence, Counseling, & Research**

**Principal Investigator:** **Sonjia Kenya, EdD, MS, MA**

**Sponsor: NIMHD**

**National Clinical Trial (NCT) Identified Number: NCT04663152**

**Version Number: 20241108**

Table of Contents

[STATEMENT OF COMPLIANCE 3](#_Toc175489879)

[1 PROTOCOL SUMMARY 3](#_Toc175489880)

[1.1 Synopsis 3](#_Toc175489881)

[2 INTRODUCTION 4](#_Toc175489882)

[2.1 Study Rationale 2](#_Toc175489883)

[2.2 Background 2](#_Toc175489884)

[2.3 Risk/Benefit Assessment 3](#_Toc175489885)

[2.3.1 Known Potential Risks 3](#_Toc175489886)

[2.3.2 Known Potential Benefits 3](#_Toc175489887)

[3 STUDY DESIGN 3](#_Toc175489888)

[3.1 Overall Design 3](#_Toc175489889)

[4 STUDY POPULATION 4](#_Toc175489890)

[4.1 Inclusion Criteria 4](#_Toc175489891)

[4.2 Exclusion Criteria 4](#_Toc175489892)

[4.3 Screening &screen Failures 4](#_Toc175489893)

[4.4 Setting 5](#_Toc175489894)

[4.5 Strategies for Recruitment and Retention 5](#_Toc175489895)

[5 STUDY INTERVENTION 6](#_Toc175489896)

[5.1 Study Intervention Description 6](#_Toc175489897)

[5.1.1 Enrollment 6](#_Toc175489898)

[5.1.2 CHW Intervention 7](#_Toc175489899)

[5.1.3 Compensation 9](#_Toc175489900)

[5.2 Measures to Minimize Bias: Randomization 10](#_Toc175489901)

[5.3 Study Intervention Compliance 10](#_Toc175489902)

[6 SUBJECT DISCONTINUATION/WITHDRAWAL 10](#_Toc175489903)

[6.1 Subject Discontinuation/Withdrawal from the Study 10](#_Toc175489904)

[6.2 Lost to Follow-Up 11](#_Toc175489905)

[6.2.1 Incarcerated Participants 11](#_Toc175489906)

[7 STUDY ASSESSMENTS 11](#_Toc175489907)

[7.1 Study Assessments 11](#_Toc175489908)

[7.2 Adverse Events and Serious Adverse Events 12](#_Toc175489909)

[7.2.1 Definition of Adverse Events (AE) 12](#_Toc175489910)

[7.2.2 Definition of Serious Adverse Events (SAE) 12](#_Toc175489911)

[7.2.3 Classification of an Adverse Event 13](#_Toc175489912)

[7.2.4 Time Period and Frequency for Event Assessment and Follow-Up 14](#_Toc175489913)

[7.2.5 Adverse and serious adverse Event Reporting 14](#_Toc175489914)

[8 STATISTICAL CONSIDERATIONS 14](#_Toc175489915)

[8.1 Statistical Hypotheses 14](#_Toc175489916)

[8.2 Sample Size Determination 15](#_Toc175489917)

[8.3 Statistical Analyses 15](#_Toc175489918)

[8.3.1 General Approach 15](#_Toc175489919)

[8.3.2 Analysis of the Primary Efficacy Endpoint(s) 16](#_Toc175489920)

[8.3.3 Analysis of the Secondary Endpoint(s) 16](#_Toc175489921)

[9 SUPPORTING DOCUMENTATION AND OPERATIONAL CONSIDERATIONS 17](#_Toc175489922)

[9.1 Regulatory, Ethical, and Study Oversight Considerations 17](#_Toc175489923)

[9.1.1 Informed Consent Process 17](#_Toc175489924)

[9.1.2 Confidentiality and Privacy 17](#_Toc175489925)

[9.1.3 CHW Training 18](#_Toc175489926)

[9.1.4 Quality Assurance and Quality Control 19](#_Toc175489927)

[9.1.5 Data Handling and Record Keeping 19](#_Toc175489928)

[9.1.6 Protocol Deviations 20](#_Toc175489929)

[9.1.7 Conflict of Interest Policy 20](#_Toc175489930)

[9.1.7 Dissemination Policy 24](#_Toc175489930)

# STATEMENT OF COMPLIANCE

The trial will be conducted in accordance with International Conference on Harmonization Good Clinical Practice (ICH GCP) and applicable United States (US) Code of Federal Regulations (CFR). The Principal Investigator will assure that no deviation from, or changes to, the protocol will take place without prior documented approval from the Institutional Review Board (IRB), except where necessary to eliminate an immediate hazard(s) to the trial subjects. All personnel involved in the conduct of this study have completed Human Subjects Protection and ICH GCP Training.

### Institutional Review Board

The Human Subject Research Office (HSRO) at the University of Miami Institutional Review Board (305-243-3195) considers the risks and benefits of research to determine whether the research should happen. The protocol, informed consent form(s), recruitment materials, and all subject materials will be submitted to UM IRB for review and approval. Approval of both the protocol and the consent form must be obtained before any subject is enrolled. Any amendment to the protocol will require review and approval by the IRB before the changes are implemented to the study. All changes to the consent form will be IRB approved; a determination will be made regarding whether a new consent needs to be obtained from subjects who provided consent, using a previously approved consent form.

# PROTOCOL SUMMARY

## Synopsis

| **Title:** | Integrated Navigation Services for Treatment Adherence, Counseling, And Research |
| --- | --- |
| **Study Description:** | The goal of this research is to determine whether a Community Health Worker (CHW) intervention including a motivational interviewing component can help achieve long term viral suppression among Black people with poorly controlled HIV. |
| **Objectives:** | The proposed project, “Integrated Navigation Services for Treatment Adherence, Counseling, and Research” (INSTACARE: R01MD018187) will evaluate the effect of a culturally-tailored intervention that integrates CHWs into HIV clinical care teams to support improved viral suppression among vulnerable PLH. This multilevel randomized controlled trial will examine the effects of 12-months of CHW support on HIV viral load among 300 Black people living with HIV.  *Note: The INSTACARE R01 builds upon the data gathered in the two-phase pilot R56 award (R56NR019755-01), which used patient, provider, and stakeholder input to develop a culturally-tailored intervention that integrates CHWs into the HIV clinical care teams to support improved viral suppression among vulnerable PLH. As a reminder, Phase 1 was a qualitative assessment of 20 stakeholders (i.e., Black PLH, caregivers, and HIV providers) to address socioeconomic barriers and perspectives on clinic-based CHW support. Phase 2 developed an implementation strategy for clinic-based CHWs from the Phase 1 qualitative results.* |
| **Endpoints:** | HIV Viral Suppression (VL<200 copies/mL) @ 12-months |
| **Study Population:** | Black Adults Living w HIV, 18+ years |
| **Phase:** | N/A |
| **Study Duration:** | 5-years |
| **Subject Duration:** | 12-months |
| **Sponsor:** | National Institute on Minority Health and Health Disparities |
| **Program Official** | Yewande A Oladeinde; [yewande.oladeinde@nih.gov](mailto:yewande.oladeinde@nih.gov) |

# INTRODUCTION

## Study Rationale

The Integrated Navigation Services for Treatment Adherence, Counseling, and Research (INSTACARE) study is a 5-year Research Project Grant (R01) funded by the National Institute on Minority Health and Health Disparities (NIMHD). INSTACARE aims to include community health workers (CHWs) as formal members of the HIV clinical care team to help improve access to healthcare and health outcomes for Black people living with HIV (BPLH). In particular, INSTACARE will examine if 12-months of CHW support can improve treatment adherence and increase viral suppression among BPLH.

CHWs trained in motivational interviewing will be embedded into the HIV clinic system to address the social determinants and individual-level factors preventing BPLH from achieving optimal health outcomes. Using a randomized controlled trial study design, 300 Black people living with HIV (PLH) will receive a 1-year CHW intervention or usual HIV care; after 12-months, healthcare metrics including viral load, medication adherence, hospitalizations, and self-efficacy in treatment adherence will be determined. The goal is to contribute to knowledge on the utility of clinic- and community- based CHWs to improve HIV outcomes among Black PLH. If successful in optimizing HIV health outcomes, this research could make a substantive contribution towards reducing other health disparities in this population.

## Background

Miami-Dade County, Florida is an HIV epicenter where Black adults account for 17% of the County’s residents and 64% of AIDS-related deaths. These disparities stem from social determinants of health (SDOH) associated with disproportionately high rates of poverty, homelessness, and unemployment. Defined as the modifiable circumstances in which people grow, live, work, and age, the SDOH are rooted in interconnected systems that have historically fostered racial discrimination, thereby limiting access to, and continuity of, care among Black populations. Compared to non-Black persons, Miami’s Black residents account for nearly 60% of the homeless, are more than twice as likely to live below the federal poverty level and are three times more likely to be unemployed. Additionally, 1 in 3 local Black persons are foreign-born, and exhibit culturally distinct HIV risk behaviors from African Americans. These barriers to care continuity and optimal HIV outcomes highlight the critical need to develop multi-level interventions to ameliorate the impact of poor SDOH on this population.

For over 15 years, the University of Miami has supported community health worker (CHW) studies to reduce disparities among Black people with HIV (PLH). Our pilot research found Black PLH who received 12 months of community based CHW support addressing SDOH achieved improved viral suppression compared to those with standard HIV care. Recently, Boston University extended this work with an observational study that illustrated clinic based CHWs trained in Motivational Interviewing (MI) improved HIV outcomes in a limited sample of Black men. However, less evidence exists about CHW interventions that provide HIV and SDOH support in both community and clinic settings. Further, the effects of this approach among a broader sample of Black PLH, including women, sexual minorities, and Black subpopulations, has not been fully examined.

The Integrated Navigation Services for Treatment Adherence, Counseling, and Research (INSTACARE) study proposes to address SDOH, system-level factors, and individual self-care behaviors among Miami’s diverse Black PLH by broadening CHW support to both community and clinical settings. MI-trained CHWs will be embedded into HIV clinical care teams, providing clinic- and community-based support to increase viral suppression among Black people with uncontrolled HIV (viral load > 200 copies/ml).

## Specific Aims


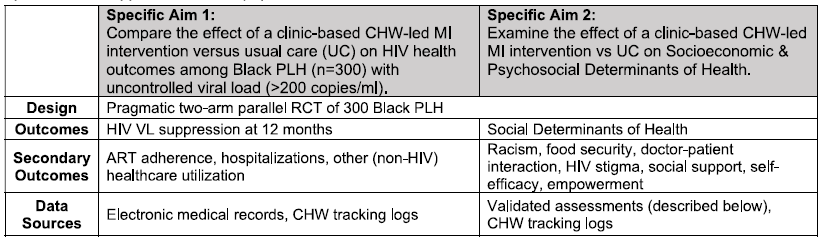


## Risk/Benefit Assessment

### Known Potential Risks

Potential risks include emotional distress and breach of privacy/confidentiality. It is possible someone not involved in this study could access patient information, or patient confidentiality may be breached from their participation in this study. As a clinic and community-based intervention, it is possible that someone from the community may observe a participant receiving care. To minimize risks these risks, CHWs are instructed to dress in a manner that is culturally consistent with norms within each participant’s neighborhood. Specifically, CHWs do not wear scrubs or any type of clothing that indicates they work in the healthcare field. Further, CHWs do not wear their clinic id badges in the field, as this could be used to associate them with a healthcare system. It is also possible that a participant could become emotionally distressed during counseling. If this occurs, the study team will refer the participant to mental health services at the healing place, a Jackson Health System mental health clinic focused specifically on PLH. It is possible that privacy regarding study participation could be breached if a participant’s phone is lost stolen or taken without their explicit consent. To minimize these risks, study team members will not leave any specific study-related information in voicemails or text messages; they will also confirm they are speaking with the participant prior to any appointment scheduling over the phone. Building social support is one-way CHWs will help participants. However, before a CHWs meets anyone in a participant’s social network, they will discuss whether the participant is comfortable explaining the CHWs role to their support system. Among participants who not comfortable disclosing their relationship with their CHW, CHWs will discuss alternative approaches to integrating social support without disclosing the participant’s study participation.

### Known Potential Benefits

Participants may receive direct benefits from participating in this study. They will receive 12-months of CHW support to engage in HIV care and treatment adherence. CHWs will work with the case managers, accompany patients to appointments, and attend clinical meetings, when possible, to provide updates on patients. In this setting, CHWs will help inform the healthcare team of areas of concern and help the team create more focused and effective individualized interventions enlightened by information CHWs have learned from non-clinical visits with participants as part of their own community-informed approaches to health promotion.

# STUDY DESIGN

## Overall Design

INSTACARE is a pragmatic two-arm parallel RCT of 300 Black PLH with unsuppressed VL receiving care at JHS Adult HIV Outpatient or Community Clinics. Within one week of enrollment, participants will have completed a baseline assessment and been randomized into the intervention arm (CHW) or usual care (UC) group. The primary outcome is viral suppression at 12-months.

**
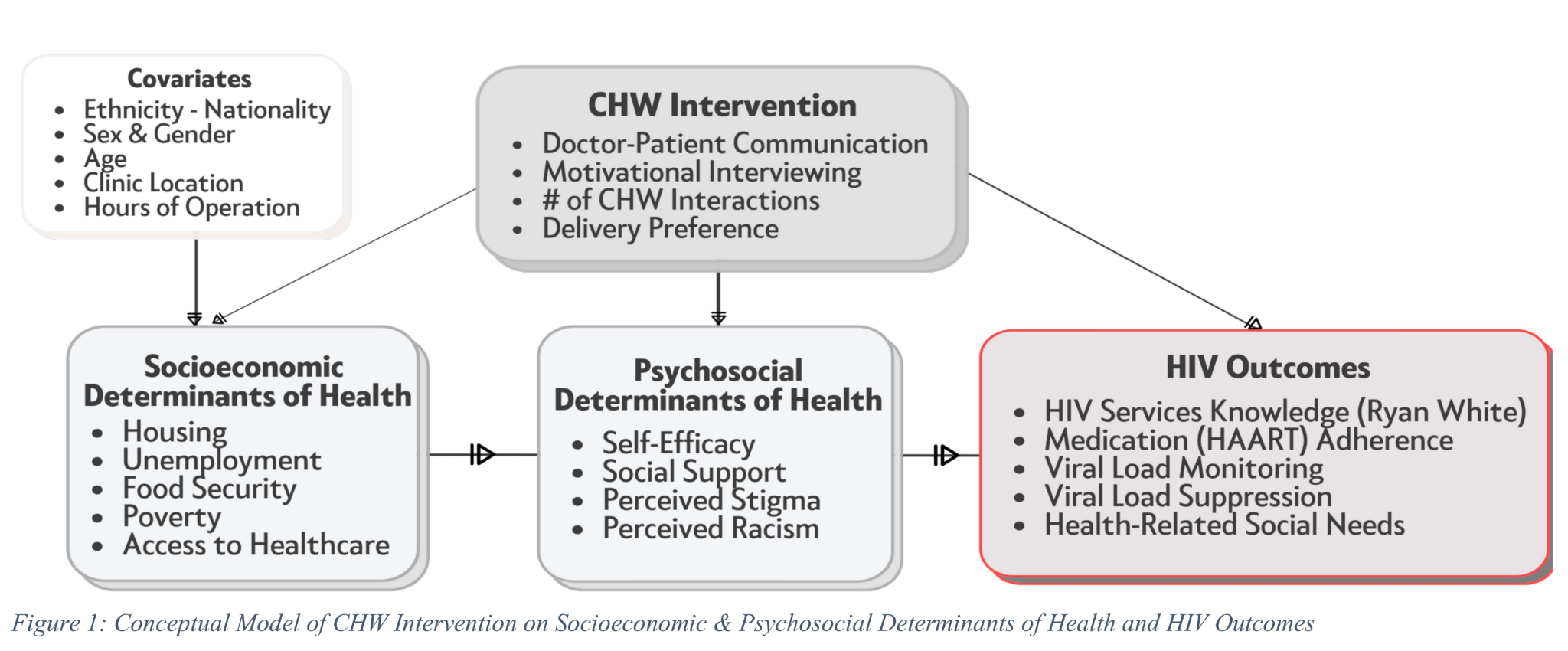
**

# STUDY POPULATION
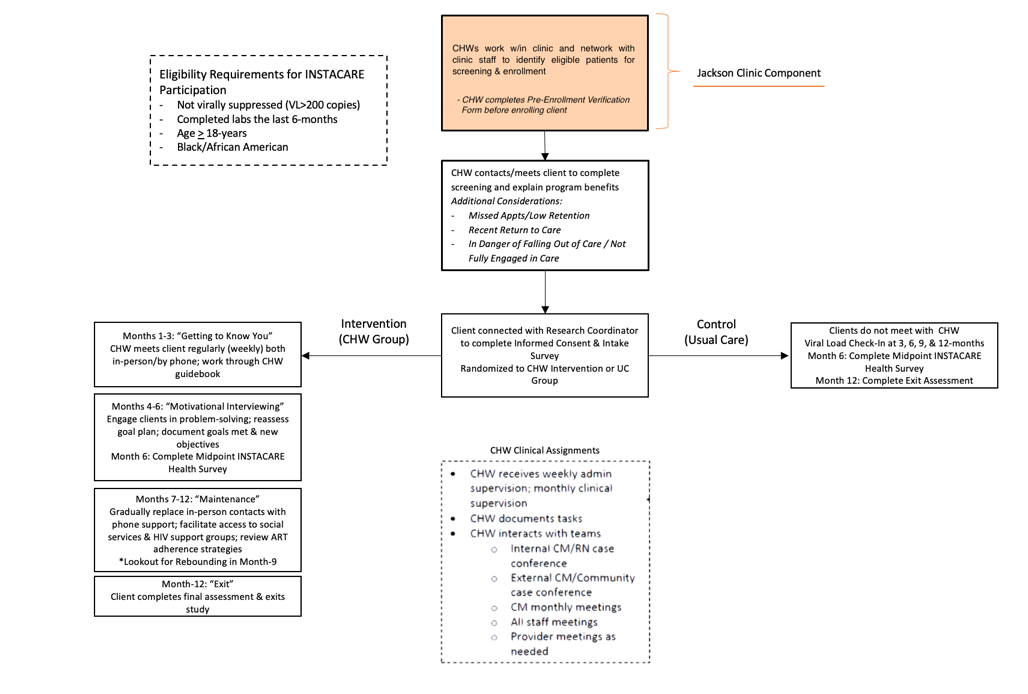


## Inclusion Criteria

Potential participants (Potentials) must meet all of the following criteria to be enrolled in the study:

- Miami-Dade Resident at time of enrollment

- **Black**/African American

- **18-years** or older

- Viral load > 200 mL within the **last 6-months** (VL must be verified via electronic medical record or lab report prior to enrollment)

## Exclusion Criteria

Potentials will be excluded from enrollment if they meet one or more of the following criteria:

- Live outside Miami-Dade County at the time of enrollment
- Do not self-identify as Black
- Under the age of 18
- Labs indicate 6-months of sustained viral suppression at the time of enrollment
- Member of a vulnerable population, including incarcerated persons; persons deemed by providers to have significant cognitive impairment/dementia; or individuals presenting with some other indication to prevent participation

## Setting

INSTACARE is based at the University of Miami Don Soffer Clinical Research Center; INSTACARE’s base location shares a campus with JHS’ flagship hospital, **Jackson Memorial Hospital**. JMH is the County’s largest safety-net hospital, staffed by interdisciplinary treatment team of faculty and clinicians from both JHS and UM. Providers note that over 750 patients living with HIV have not achieved viral suppression and many patients face significant barriers to care including extreme poverty, homelessness, alcohol and substance use, mental illness, domestic violence, and other social challenges that impact medication adherence. Recruitment will begin at this site.

Information about the INSTACARE study and our project goals will also be presented to local clinics throughout Miami-Dade County to enhance community engagement efforts and increase awareness about the study. Recruitment materials will also be shared with these local agencies to facilitate promotion of the program and encourage interested potentially eligible patients to contact the study team for more information. The following HIV care agencies will be contacted for referral support: Jessie Trice, Empower U, Care Resource, Borinquen Medical Centers, AIDS Healthcare Foundation, Pridelines, Care 4 U, Community Health of South Florida, Better Way, Latinos Salud, and the following Jackson Health System locations:

- Dr. Rafael Peñalver Clinic
- Jackson North Medical Center
- Jackson South Medical Center
- Jackson West Medical Center
- Jefferson Reaves Health Center
- Jose Milton Memorial Hospital
- North Dade Health Center
- Prevention Education & Treatment Center
- SFAN – Hialeah
- SFAN – Florida City

These sites will not be engaged in the research project; they will only facilitate in the participant referral process (e.g., displaying study flyers, sharing brochures with potentially eligible clients, etc.).

## Strategies for Recruitment and Retention

Recruitment for INSTACARE is referral-based, which could include: 1) a clinic or community health center; 2) a physician or health provider; or 3) self-referral. Eligible clients are identified by their provider through the patient’s medical records. To obtain referrals, CHWs can use the following recruitment strategies:

1. Jackson Clinic Attendance: Clinic attendance involves regular communication – in-person, by phone, via email/SMS messaging, etc. – with clinical staff (physicians, nurses, case manager) to identify eligible patients. CHWs must ensure clinic staff are informed of our study eligibility and be prepared for potential same-day enrollment among walk-in patients, when feasible.
2. Collaboration with Community Stakeholders & HIV Care Teams: CHWs are encouraged to identify, network, and collaborate with other HIV agencies, community stakeholders, and ID research programs to identify programs with overlapping patient populations. For example:
   1. Biweekly contact with ID Research staff to review new cases enrolled in JHS research database
   2. Study brochure distribution and program education at local centers, community events, etc. to increase word-of-mouth engagement and referrals
   3. Attending FDOH provider meetings (e.g. Florida Black HIV/AIDS Coalition)

Contacting Potential Participants: Potential participants may indicate interest in participating but be unavailable for same day enrollment. CHWs should follow up with the identified potential participants by phone within one week of initial referral [See INSTACARE Phone Script for exact language to be used during recruitment phone calls]. CHWs should make 3 attempts to contact the potential. The potential participant must be re-referred by a provider for additional contact attempts.

# STUDY INTERVENTION

## Study Intervention Description

### Enrollment

**Setting**

- ***Office/Clinic Enrollment***: Enrollment is traditionally conducted at the INSTACARE offices, located in the Don Soffer Clinical Research Center on the Miller School of Medicine campus. When available, enrollment can also be completed at the Infectious Disease Research Unit within Jackson Memorial. This location is subject to availability based on the ID Unit Appointment Schedule (reservations are preferred).
- ***In-Field Enrollment***: Enrollment can also be performed off-campus at a mutually agreed public location. CHWs are encouraged to use the buddy system, when possible, for field-based enrollment. Staff electronic devices (including mobile devices and tablets) are equipped with cellular hotspots and unlimited data to facilitate electronic data collection. CHWs are encouraged to identify locations with ample cell service when feasible.

**Materials**

- ***CRC Enrollment Materials***: Prior to enrollment at one of our CHAMP offices, CHWs should ensure they have the following printed documentation available:

1. Informed Consent Form (in the clients preferred language)
2. W-9 Form
3. Receipt Page
4. Medical Release Form
5. ClinCard (in sealed envelope)

*Items 2-4 must be signed by the participant for our records.

- ***In-Field Enrollment Materials***: In addition to the documents above, CHWs should also have the following documents printed for field-based enrollment:

1. Intake Information Form
2. INSTACARE Health Survey

If you are unable to access the online database, CHWs should be prepared to complete the data collection forms on paper.

**Enrollment Procedures**

*Note: All enrollment procedures should be conducted in person.*

- **Data Entry Procedure**

1. Log into REDCap, select “My Projects” from the header, and select ”[INSTACARE: Integrated Navigation Services for Treatment Adherence, Counseling, and Research](https://redcap.miami.edu/redcap_v14.0.12/index.php?pid=7479)”
2. Select Add/Edit Records from the menu on the lefthand side of the page
3. Enter the new Client ID and press enter
4. Complete all documents listed under ***Baseline Enrollment***:
   1. **Informed Consent**
      1. **For Spanish- or Kreyol-speaking clients**: a member of the research team is available to translate the consent form for the client’s comfort. Note: Patients must speak/understand basic English to be eligible for enrollment.
      2. **Client unable to provide digital signature on REDCap**: If a participant is unable to digitally sign the informed consent form during the enrollment process, they must sign a paper copy of the ***Informed Consent Form*** for our records. The CHW should sign the electronic record and enter the following field note: “Client signature captured on file.”
   2. Intake Information
   3. INSTACARE Health Survey

- For each form, ensure all questions are completed. If so, at the end of the page, you will select “Complete” at the question called “Form Status”. The click “Save & exit form.”
- Once the INSTACARE Health Survey is complete, the CHW must contact the research coordinator for randomization. RC will indicate if the participant was assigned to the intervention or the control group (usual care).
- Clients randomized to the intervention will be assigned based on demographics (e.g. Kreyol speaking, etc.) and current CHW workload. Half of all participants will be assigned to the Usual Care group.

1. Provide payment ($125 for baseline) to participant via [ClinCard](https://miamiedu.sharepoint.com/:b:/r/sites/CHAMPSTRONG/Shared%20Documents/General/INSTACARE/ClinCard/ClinCard%20Reference%20Guide%20-%20UM%20Site%20Coordinator%205.21.pdf?csf=1&web=1&e=akAvmv) System
   1. Complete W-9; have client sign paper receipt
   2. Participants randomized to the CHW group will be assigned to a CHW within one month of initial *referral* and within one week of enrollment.

### CHW Intervention

Interactions between CHWs and patients will occur through a) clinic visits, b) home visits, and c) phone or mobile contact.

*Note: Refer to the INSTACARE (COACH) Workbook for CHW guide

- **Baseline “Getting to Know You”**: The baseline patient evaluation is estimated to take 90 minutes.

Within one week of being assigned a patient, CHWs will schedule a meeting to perform a comprehensive, face-to-face evaluation of the participants' socioeconomic, social support, and healthcare barriers to ART adherence, including:

- Stability of housing, food, immediate resources, social support, immigration status, life stressors
- Barriers to interacting with the healthcare system such as communicating with providers, transportation needs, insurance status, and unmet mental health needs
- Understanding HIV and HIV medications
- Knowledge of their own VL (patient ability to self-monitor VL changes) Current lifestyle behaviors, including use of cigarettes, alcohol and/or non-prescribed drugs, and transmission risk behaviors (e.g. condom use, disclosing HIV status to partners, multiple sexual partners)
- Communication preferences, (e.g. mobile phone knowledge and usage)

*Note: While INSTACARE is focused on improved VL through increased ART adherence, CHWs also address these issues from an overall health and general well-being approach.

- **Individual Action Plan**: After the evaluation, review each participant's unique circumstances and work with the client to design an individualized plan to overcome barriers to ART adherence, beginning with the most pressing needs.
- **Months 1-3**
  1. Conduct an average of one face-to-face weekly visit to provide acute support focused on HIV management and HIV education for the first month of enrollment. Face-to-face meetings can gradually be replaced with phone calls beginning in Month-2 depending on the severity of your clients’ needs
  2. Accompany participants to the majority of their medical appointments and review treatment plans to ensure they understand provider recommendations.
  3. Based on individual needs and preferences, engage participants in problem-solving processes to set priorities for immediate problem resolution
  4. Set personal health goals regarding VL control (i.e., improved ART adherence, addressing barriers to care, resolving ambivalence about HIV treatment)
  5. Develop a plan to accomplish those goals and review results
  6. At the end of Month-3, participants should have a follow-up visit scheduled with their provider for routine viral load testing. If not, help the participant schedule an appointment; collect viral load data from EMR to add to their INSTACARE record.
  7. Participants will receive $50 after completing the 3-month assessment. Payment will be loaded to their ClinCard (receipt automatically recorded online @ Greenphire ClinCard website)
- **Months 4-6**

***Strengthening Self-Efficacy to Manage HIV***

- 1. Depending on each participant's development, in-person meetings will gradually be replaced with phone-based support as they improve skills to manage HIV.
  2. Continue to review personal health goals regarding VL control (i.e., improved ART adherence, addressing barriers to care, resolving ambivalence about HIV treatment)
  3. Review plan to accomplish those goals and discuss client results
  4. Develop new personal goals as needed
  5. By Month-5, contacts should largely be made by phone (not including clinic appointments or other support services)
  6. At the end of Month-6, participants should have a follow-up visit scheduled with their provider for routine viral load testing. If not, help the participant schedule an appointment; collect viral load data from EMR to add to their INSTACARE record.
  7. Participants also complete their 6-month Assessment. The 6-month assessment will take approximately 45 minutes to complete.
  8. Participants will receive $50 after completing the 6-month assessment. Payment will be loaded to their ClinCard (receipt automatically recorded online @ Greenphire ClinCard website)

***Important HIV Management components include:***

- - - - CHW counseling/coaching
      - Medical service support/patient navigator activities: help patients with appointment scheduling/reminders, navigating health system bureaucracy, identifying strategies to maximize their time with the providers
      - Goal setting for VL
      - Individual lifestyle interventions: using MI strategies based on Stages of Change model of behavior counseling
      - Referral to existing community-based resources that may be socially or medically based: tenant advocacy, immigration legal services, domestic violence programs, mental health programs, smoking or substance abuse, etc.

*Note: If agreeable, patient, family and caregiver involvement in these sessions will be highly encouraged. The ultimate goal of these activities is for participants to develop and/or improve self-efficacy and social support to manage their HIV health beyond intervention participation.

- **Months 7-12**

***Maintenance:*** *After the first six months, participants will enter the Maintenance Phase.*

- 1. Contact participants 2-3x per month for check-ins on client status (medication adherence, social needs, etc.); in-person interactions on an as needed basis. Contacts include appointment reminders, facilitating contact with providers, and rescheduling appointments as needed.
  2. Check for rebounding sixty (60) to 90 days before study exit & facilitate access to additional social services and HIV support groups as needed.
     - ***Note***: *Some participants may require intensive face-to-face support in Month-9. Participants at this stage are at risk for “****rebounding****,” where their viral load begins to increase again. Additional attention may be needed to maintain progress.*
     - Discuss these cases with the Pl and Co-I (Dr. Rodriguez) who will approve any changes, such as modifying service intensity or altering the type of support delivered (i.e., review ART adherence strategies, as well as the process of maintaining health insurance and social service benefits, including food stamps, housing assistance, transportation vouchers, and mental health support)
     - E.g., The Adult Clinical Immunology Education Initiative provides psychological guidance to PLH.
  3. As the 12-month study period nears conclusion, ensure participants are linked participants to community- and clinic-based social support resources.

### Compensation

Participants will receive $400 for taking part in this study – $125 at enrollment and exit, and $50 at the 3-, 6-, and 9-month viral load assessments. Compensation will be offered using ClinCard. ClinCard is a MasterCard-branded debit card that can be loaded with funds by the study team. The card will be loaded according to the payment schedule (enrollment, 3-, 6-, 9-, and 12-months).

This card is administered by a company called Greenphire. Greenphire requires name, address and date of birth entered online for payment; mobile phone number and/or email address are optional and only for study related communications. This information will only be used for payment and communication purposes and will not be given to another company or linked to any of the study data. Greenphire will not receive any information about client health status or the study in which they are participating.

If the card is lost or stolen, participants are instructed to call the study team for a replacement card. Participants are further instructed that losing the card is similar to losing cash; any money previously loaded on the card cannot be recovered.

Some participants may be eligible for cash payments. These cases are rare and may be offered based on the study team’s judgement.

## Measures to Minimize Bias: Randomization

Randomization is a process that assigns participants/subjects by chance (rather than by choice) into specific groups, typically for clinical research and clinical trials.

The goals of randomization include:

- - - 1. To produce groups that are comparable (balanced)
      2. To remove bias (selection bias)
      3. To guarantee the validity of statistical tests

INSTACARE follows a stratified block randomization structure. Block randomization is designed to ensure a balance in sample size across groups over time. Blocks are small and balanced with predetermined group assignments, which keeps the numbers of subjects in each group similar at all times. Block size should be a multiple of the number of groups. INSTACARE has two study arms, or groups: the CHW (intervention) group and the Usual Care (control) group. The groups are stratified by sex (male, female) with a block size of 6. Subject allocation is implemented using the randomization module in REDCap, which also monitors the overall allocation progress and assignment of randomized subjects. As a minimal risk study, the investigative team does not anticipate the need to unblind the trial due to patient safety concerns or serious adverse events. As a precaution, the project statistician can be unblinded to the randomization through REDCap. The PI can use REDCap’s Randomization Dashboard to view the overall allocation progress and assignments for subjects that have been randomized. Subjects are randomized by a research coordinator blinded to the sample.

## Study Intervention Compliance

Participants have up to 6-weeks after their scheduled follow-up date to complete labs. After 6-weeks, the entry will be marked as MISSED. Participants will not be compensated for missed F/U appointments.

Participants in the control group will receive a call at least two weeks before their quarterly follow-up to remind them to complete their labwork and/or survey. Clients can complete labs up to six weeks past their follow-up due date. For the 3- and 9-Month follow-up appointments, CHWs can attempt to contact participants weekly for up to 6-weeks after the due date. At the 6- and 12-Month F/U appointments, CHWs will attempt to reach all participants by phone to schedule and complete their survey which can be in person or by phone. Attempts to contact participants by phone should occur at different times and days of the week including evenings and weekends. Those that cannot be reached should also have a letter delivered to their address on file (when feasible) asking them to call the study team.

# SUBJECT DISCONTINUATION/WITHDRAWAL

## Subject Discontinuation/Withdrawal from the Study

Participants are free to withdraw from participation in the study at any time upon request. However, we seek to minimize participant withdrawal from the study by ongoing engagement as described above for the intervention group.

Clients seeking to withdraw from the study can be asked to complete the exit assessment and can receive $50 compensation for completing the assessment at time of withdrawal.

Clients who sign the informed consent form and are randomized but do not receive the study intervention may be replaced. Participants who sign the informed consent form, and are randomized and receive the study intervention, and subsequently withdraw, or are withdrawn or discontinued from the study, will not be replaced.

## Lost to Follow-Up

A participant will be considered lost to follow-up if he or she is unable to be contacted by the study site staff for 3-months. Before a participant is deemed lost to follow-up, the staff will make every effort to regain contact with the participant (where possible, weekly phone calls, calling/visiting emergency contacts, performing house visits, and if necessary, a delivering a letter to the subject’s last known mailing address or local equivalent methods). These contact attempts should be documented in the client’s study file.

After 3-months, the client will be considered **Lost to Follow-Up**. Additional contact attempts made by the CHWs will be limited to one quarterly phone call/voicemail reminding clients of upcoming lab/assessment dates. Lost to Follow-Up clients will not be withdrawn from the study, as findings from our pilot intervention suggest clients may return seeking support at a later date. Participants that resurface within the 12-month intervention period can continue to receive support until their scheduled exit date.

### Incarcerated Participants

The National Institutes of Health has policies that require additional safeguards for certain vulnerable populations. These safeguards are particularly important for incarcerated populations as they may not be able to make a truly voluntary and uncoerced decision regarding their participation in research. CREW is also limited in its ability to contact clients for follow-up services or to compensate clients for time while incarcerated. Regulations for prisoners further include requirements for a prisoner representative on the IRB and certification by the study by the HHS Secretary.

As a result of these regulations, clients that become incarcerated after enrollment in the study will automatically be deemed lost to follow-up. CHWs should check public records at least once a quarter to determine changes in sentencing, release, and/or parole.

# STUDY ASSESSMENTS

## Study Assessments

Medical data, including HIV viral load, from the 300 participants will be obtained from the electronic medical records. The study team will maintain a separate, secure database at UM having information on participant age, race, ethnicity, ZIP code, and group assignment. Additional data on several areas related to HIV management, including medication adherence and self-efficacy will also be collected. Data will periodically be transferred from UM’s secure database to REDCap, UM’s web-based clinical research management application for clinical and translational research. Below lists the assessments that will be used for patient tracking, as well as the CHW training evaluation.

| **Specific Aim 1: Primary Measures** | | Durable HIV viral suppression prevents forward transmission of HIV, making HIV treatment synonymous with HIV prevention. The primary outcome is binary: HIV viral suppression (plasma HIV VL of < 200 copies/mL) at the 12-month follow-up. As part of usual medical care, patients have VL checked every 3 months. Thus, data on the primary outcome will be abstracted from the electronic medical record by a study coordinator. All patients who are not up to date on VL measurements will be encouraged to go to their assigned laboratory (JHS, Quest or LabCorp) to have the test done. By Month-11 of enrollment, the staff will call participants to ensure Exit is scheduled on time. |
| --- | --- | --- |
| Viral Load Suppression | Plasma HIV RNA  (>200 copies / mL) |  |
| **Specific Aim 1: Secondary Measures** | |  |
| ART Adherence | Medication adherence (ACTG Adherence Questionnaire) |  |
| Non-HIV Medical Care | Medical Records |  |
| Hospitalizations | Medical Records |  |

| **Specific Aim 2: Primary Measures** | |
| --- | --- |
| ***Structural*** | |
| Social Determinants of Health | Health-Related Social Needs (HRSN) |
| Racism | Index of Race-Related Stress-Brief (IRRS-B) |
| Food Security | Household Food Insecurity Access Scale (HFIAS) |
| ***Institutional*** | |
| Dr-Pt Interaction | Doctor-patient communication questionnaire: empathy, decision-making, listening |
| HIV Stigma – Clinic Setting | Perceived Discrimination Scale: Discrimination, mistreatment, quality of care, & mistrust from healthcare system |
| ***Community*** | |
| HIV Stigma – Community Setting | HIV Stigma Scale – Short Version: Perceptions of stigma, disclosure concerns, concerns with public attitudes and negative self-image |
| ***Interpersonal*** | |
| Social Support | Frequency of & satisfaction with social support |
| ***Individual*** | |
| Health Outcomes | Medical Outcomes Study HIV Health Survey (MOS-HIV): Quality of life measure for PLH |
| Self-Efficacy | HIV Adherence Self-Efficacy Scale (HIV-ASES): social & psychological determinants of adherence |
| Empowerment | Health Empowerment Inventory: Patient-controlled engagement and involvement in healthcare |
| **Specific Aim 2: Secondary Measures** | |
| Motivational Interviewing | CHWs’ MI training and retention will be measured with the Motivational Interviewing Treatment  Integrity Code (MITI 4), administered by a trained MI expert |
| CHW Delivery Strategies | Participant preferences will be extracted from CHW tracking logs to perform a frequency analysis of delivery preferences. Communication (phone, text, in-person); Setting (clinic, community, home); and Support (education, navigation, social support linkage) |

## Adverse Events and Serious Adverse Event

### Definition of Adverse Events (AE)

Adverse event means any untoward medical occurrence associated with the use of an intervention in humans, whether or not considered intervention-related (21 CFR 312.32 (a)).

### Definition of Serious Adverse Events (SAE)

An adverse event (AE) is considered “serious” if, in the view of either the investigator or sponsor, it results in any of the following outcomes:

- Death
- A life-threatening adverse event (of note, the term “life-threatening” refers to an event in which the subject was at risk of death at the time of the event, rather than to an event which hypothetically might have caused death if it were more severe)
- inpatient hospitalization or prolongation of existing hospitalization
- a persistent or significant incapacity or substantial disruption of the ability to conduct normal life functions
- or a congenital anomaly/birth defect.

Important medical events that may not result in death, be life-threatening, or require hospitalization may be considered serious when, based upon appropriate medical judgment, they may jeopardize the subject and may require medical or surgical intervention to prevent one of the outcomes listed in this definition. Examples of such medical events include allergic bronchospasm requiring intensive treatment in an emergency room or at home, blood dyscrasias or convulsions that do not result in inpatient hospitalization, or the development of drug dependency or drug abuse.

### Classification of an Adverse Event

#### Severity of Event

For adverse events (AEs), the following guidelines will be used to describe severity:

- **Mild** – Events require minimal or no treatment and do not interfere with the subject’s daily activities.
- **Moderate** – Events result in a low level of inconvenience or concern with the therapeutic measures. Moderate events may cause some interference with functioning.
- **Severe** – Events interrupt a subject’s usual daily activity and may require systemic drug therapy or other treatment. Severe events are usually potentially life-threatening or incapacitating. Of note, the term “severe” does not necessarily equate to “serious.”

#### Relationship to Study INTERVENTION

All adverse events (AEs) must have their relationship to study intervention assessed by the clinician who examines and evaluates the subject based on temporal relationship and his/her clinical judgment. The degree of certainty about causality will be graded using the categories below. In a clinical trial, the study product must always be suspect.

- **Related** – The AE is known to occur with the study intervention, there is a reasonable possibility that the study intervention caused the AE, or there is a temporal relationship between the study intervention and event. Reasonable possibility means that there is evidence to suggest a causal relationship between the study intervention and the AE.
- **Not Related** – There is not a reasonable possibility that the administration of the study intervention caused the event, there is no temporal relationship between the study intervention and event onset, or an alternate etiology has been established.

#### Expectedness

The Principal Investigator will be responsible for determining whether an adverse event (AE) is expected or unexpected. An AE will be considered unexpected if the nature, severity, or frequency of the event is not consistent with the risk information previously described for the study intervention.

### Time Period and Frequency for Event Assessment and Follow-Up

The occurrence of an adverse event (AE) or serious adverse event (SAE) may come to the attention of study personnel during study visits and interviews of a study subject presenting for medical care, or upon review by a study monitor.

All AEs including local and systemic reactions not meeting the criteria for SAEs will be captured on the appropriate case report form (CRF). Information to be collected includes event description, time of onset, clinician’s assessment of severity, relationship to study product (assessed only by those with the training and authority to make a diagnosis), and time of resolution/stabilization of the event. All AEs occurring while on study must be documented appropriately regardless of relationship. All AEs will be followed to adequate resolution.

Any medical condition that is present at the time that the subject is screened will be considered as baseline and not reported as an AE. However, if the study subject’s condition deteriorates at any time during the study, it will be recorded as an AE.

Changes in the severity of an AE will be documented to allow an assessment of the duration of the event at each level of severity to be performed. AEs characterized as intermittent require documentation of onset and duration of each episode.

The Study Coordinator will record all reportable events with start dates occurring any time after informed consent is obtained until 7 (for non-serious AEs) or 30 days (for SAEs) after the last day of study participation. At each study visit, the Study Coordinator will inquire about the occurrence of AE/SAEs since the last visit. Events will be followed for outcome information until resolution or stabilization.

### Adverse and Serious Adverse Event Reporting

All serious adverse events must be reported to the IRB according to regulatory requirements. The Principal Investigator will immediately report to the sponsor any serious adverse event, whether or not considered study intervention related, including those listed in the protocol or package insert and must include an assessment of whether there is a reasonable possibility that the study intervention caused the event. Study endpoints that are serious adverse events (e.g., all-cause mortality) must be reported in accordance with the protocol unless there is evidence suggesting a causal relationship between the study intervention and the event (e.g., death from anaphylaxis). In that case, the investigator must immediately report the event to the sponsor.

All serious adverse events (SAEs) will be followed until satisfactory resolution or until the Principal Investigator deems the event to be chronic or the subject is stable. Other supporting documentation of the event may be requested and should be provided as soon as possible.

# STATISTICAL CONSIDERATIONS

## Statistical Hypotheses

**Primary Efficacy Endpoint(s):**

SPECIFIC AIM 1: Compare the effect of a clinic-based CHW-led MI intervention versus usual care (UC) on HIV health outcomes among Black PLH (n = 300) with uncontrolled viral load (>200 copies/ml). Aligned with the NIMHD Minority Framework for Health and Health Disparities Research, this pragmatic two-arm parallel RCT CHWs will support a multilevel approach to improving HIV care outcomes among Black PLH in Miami-Dade. The primary outcome is HIV viral load suppression at 12-months.

**Secondary Efficacy Endpoint(s):**

SPECIFIC AIM 2: Examine the effect of a clinic-based CHW-led MI intervention vs UC on Socioeconomic & Psychosocial Determinants of Health. Social Determinants of Health (SDOH) are drivers of health disparities and evidence demonstrates improved SDOH leads to improved health outcomes. INSTACARE’s CHW intervention aims to address the negative SDOH impacting HIV health among participants. These determinants are assessed using the validate measures included in the INSTACARE Health Survey delivered at baseline, 6-months, and study exit.

## Sample Size Determination

To estimate the sample size required for this study, we reviewed VL suppression data from our pilot study in which the CHW intervention achieved a significant VL reduction of 1.5 log10 copies/ml versus usual care (study underpowered to examine viral suppression). Based on that analysis, we hypothesize that the proportion of patients achieving viral suppression in the CHW arm will be at least twenty percentage lower than those randomized to the UC group. Using an alpha significance level of 0.05, two-sided t-test analyses estimate that 147 participants per study arm (294 participants in total) will allow us to detect this minimum difference between groups in this binary outcome with 90% power (see table in statistical analysis section). Even with 20% attrition our study would still have over 80%power to detect this difference. Thus, we plan to recruit 300 patients over a 48-month period and follow each patient for 12 months. For the proposed study, we will have a total sample size of 300 black patients with unsuppressed VL randomized (1:1) into two groups (150 per study arm) into one of two arms: (1) CHW intervention or (2) UC. From clinical discussions amongst the research team, we expect that participants in (1) will have at least a 20% higher proportion of viral suppression compared to (2). Black HIV-infected individuals who are admitted to UC usually will have low rates of viral suppression. With 300 black patients and with the assumption that the proportion of viral suppression in the control group is 25%, we will have above 91% power to detect at least a 20% absolute difference between the two treatment arms. Even with a 20% attrition rate we will still be able to detect an absolute difference as low as 17% with over 80% power. For the secondary outcomes, there will be over 80% power to uncover a standardized difference between groups of .10, which is considered a small effect. The test statistic used is the two-sided Z-Test with unpooled variance while the significance level of the test is 0.05. As for the mediation analysis, with an assumption of a relatively large ICC at 0.4, and a small effect size for both pathway of a (0.14) and b (0.14), and four observations (at baseline, 3, 6 and 12 months), 276 participants will provide 80% power using bootstrap method. While with two observations (at baseline and 12 months) and a small ICC at 0.1, 300 will also provide around 80% power with the bootstrap method.

## Statistical Analyses

### General Approach

Using a randomized parallel-controlled design, we will evaluate the effects of our CHW intervention on VL suppression among nonadherent or acutely infected PLH. Analysis will be led by Dr. Pan.

- Preliminary Data Analysis: Initial data analyses will include (a) descriptive statistics across time points for all variables and (b) testing of distributional assumptions of all variables, including statistical tests for univariate/multivariate normality and visual inspection of distributions of all variables (graphical examination of data). Should significant deviations from normality occur, transformation of variables will be attempted.

- Missing Data: Missing data are a ubiquitous problem in HIV behavioral research, primarily due to dropping out and refusal. We will make every effort to minimize missing data. During the analytic phase, we will carefully assess missing data (whether missing occurs at random). Missingness patterns will be identified, and analyses will be conducted to determine if there is differential attrition by treatment arm, and if missingness is related to any of the covariates. If nonrandom missingness is of concern, it will be addressed by applying pattern-mixture, propensity score or related models so that bias effect can be assessed in sensitivity analyses.

### Analysis of the Primary Efficacy Endpoint(s)

As specified in the aims, the primary hypothesis test will compare the proportions achieving HIV viral suppression at 12 months across the two study groups. All treatment comparisons will be performed under the Intent-to-Treat (ITT) criterion in the sense that participants will be analyzed in the arm to which they were randomized, regardless of subsequent events. Using a logistic GEE model, a type III contrast between CHW and UC arms will be estimated in a model with a binomial distribution and logit link as implemented in SAS for the primary outcome. The GEE model will include both 12-month HIV viral suppression status, and HIV viral suppression status at 3 months intervals. However, the primary test of the hypothesis will be based on contrasts involving the 12-month proportions only. This model gains statistical power by pooling the error term across the four follow-up times (3, 6, 9, and 12 months) and is closer to an ITT approach than would be the analysis of 12-month events only. Univariate and multivariate models will be used to calculate odds ratios and corresponding 95% confidence intervals for associations between viral suppression at 12 months and two treatment arms, controlling for covariates such as sociodemographics and potential confounders. In addition, stratified analysis will be examined for gender, ethnic, and immigration status differences. Two-way interaction terms between various study variables and the study arms may also be included in the models. Standard diagnostic tools will be used to assess model fit. Missing data associated with drop out or any other cause will not be imputed in the primary analyses. If needed, alternative analyses may explore others model to account for such missing data such as last valued carried forward or multiple imputation.

### Analysis of the Secondary Endpoint(s)

We will test the secondary outcomes respectively. Those secondary outcomes that are binary will be tested as described for the primary hypothesis using a logistic regression; whereas those secondary outcomes that involve either continuous or ordinal variables will utilize the appropriate distribution and link functions. Note that the exact method of analysis will depend on the realized distribution of the particular outcome in this trial. For example, an expected count data variable may need to be modeled using a zero-inflated Poisson Regression rather than a Poisson regression if there are too many zero observations to fit the standard Poisson. If there is over-dispersion, a negative binomial (or zero-inflated negative binomial) regression may be appropriate. Models will be estimated with main effects for these variables, a main effect for randomization group, and an interaction between the particular variable and randomized group on the primary outcomes. In addition, we will also conduct sensitivity analyses to explore and determine the association of specific CHW strategies, and the frequency of these strategies, with outcomes. The impact of CHW services will be measured 1) by the way care is delivered (in-person, phone, text); 2) the type of support provided (health education, health system navigation, linkage to social services, empathy), and 3) the setting in which interactions occur (community, clinic, home, virtual). A Bonferroni correction will be applied to secondary outcomes. Mediation: Mediation will be tested using structural equation modeling with Mplus 8.7. These models estimate the effect of the intervention on the potential mediators from secondary outcomes (path a) and the effect of the mediator on the viral suppression outcome or next proximal intermediate outcome (path b). Longer mediation pathways can also be tested (e.g., a*b*c). There is significant mediation if the product of these two paths (a*b) is greater than zero. Statistical significance will be assessed using bias-corrected bootstrap confidence intervals on the product terms. This test is by far the most powerful test of mediation and can test multiple mediating pathways within a single structural model.

# SUPPORTING DOCUMENTATION AND OPERATIONAL CONSIDERATIONS

## Regulatory, Ethical, and Study Oversight Considerations

### Informed Consent

#### Consent Procedures and Documentation

Informed consent is a process that is initiated prior to the individual’s agreeing to participate in the study and continues throughout the individual’s study participation. Consent forms describing in detail the study intervention, study procedures, and risks are given to the subject and written documentation of informed consent is required prior to conducting enrollment procedures.

CHWs will explain the research study to the subject and answer any questions that may arise. A verbal explanation will be provided in terms suited to the subject’s comprehension of the purposes, procedures, and potential risks of the study and of their rights as research subjects. Subjects will have the opportunity to carefully review the written consent form and ask questions prior to signing.

The subject must sign the informed consent document prior to any procedures being done specifically for the study. Subjects must be informed that participation is voluntary and that they may withdraw from the study at any time, without prejudice. A copy of the informed consent document should be offered to the subjects for their records. The rights and welfare of the subjects will be protected by emphasizing to them that the quality of their medical care will not be adversely affected if they decline to participate in this study.

### Confidentiality and Privacy

Subject confidentiality and privacy are strictly held in trust by the participating investigators and their staff. Therefore, the study protocol, documentation, data, and all other information generated will be held in strict confidence. No information concerning the study or the data will be released to any unauthorized third party without prior written approval of the Principal Investigator.

Data will be collected from the EMR or subjects at UHealth or JHS.

In compliance with HIPAA, individual subject confidentiality is assured through the use of unique study identification codes throughout the processing and analyses. Physical and electronic study subject research data used for purposes of statistical analysis and scientific reporting, will be stored at the INSTACARE Research Office based at the University of Miami Miller School of Medicine. Electronic data is stored on a University of Miami electronic device (e.g. encrypted, password-protected computer) and on a cloud-based storage system that is approved by the University of Miami (e.g. the University’s REDCap data management system, which is backed up to University servers daily). These records will not include the subject’s contact or identifying information. Rather, individual subjects and their research data will be identified by a unique study identification number. The study data entry and study management systems used by research staff will be secured and password protected. Staff are provided with University-issued computers, tablets, and mobile devices equipped with password-protection and University-issued encryption.

At the end of the study, all records will continue to be kept in a secure location for as long a period as dictated by the reviewing IRB and/or Institutional policies.

### CHW Training

**FL Department of Health HIV Counselor Training**

1. **500 & 501 Online Training:** The FDOH HIV Counselor Training includes two online courses and an “in-person” (virtual) workshop.
2. The online training modules are provided by the Florida Department of Health through the TRAIN Florida Learning System. Please create a TRAIN Florida Account at <https://www.train.org/florida/user/register> to complete the courses.
3. Once you have an account, search for **FDOH HIV 500** or [click](https://www.train.org/florida/course/1050497/compilation) here to link to the course.
4. Select the registration tab to sign up for the course sessions. The 500 course has 5 sections, plus a learner’s guide and introduction, and each section has a short quiz at the end.
5. Once you complete 500, you’ll be able to register for 501. Click [here](https://www.train.org/florida/course/1058541/compilation) to link to the 501 course.
6. The 501 course has 6 sections, plus the learner’s guide and introduction, and each section has a short quiz at the end.
7. When you finish the training, download and sign the [500/501 Participation Agreement](https://www.testmiami.org/documents/500-501-participant-agreement.pdf). Please send a copy of the agreement and your certificates of completion to the Research Cooridnator & Program Manager.
8. ***In-Person (Virtual) Workshop:*** Once you complete the two courses, you can register for the two-day virtual workshop at <https://www.testmiami.org/forms/500-501.html>.
9. Before registration, confirm the registration dates with the Program Manager via email.
10. Once the date is confirmed, email your certificates to the FDOH HIV Program Consultant (Lori Jordahl: Lori.Jordahl@flhealth.gov). Lori will then send back a link with the available The workshop is a video conference call from 9am-1pm.
11. Lori will send you a list of available dates to choose from once she’s received your 500/501 certificates. For more details on the FDOH training, click [here](https://www.testmiami.org/providers.html#:~:text=TRAININGS,501%20Participant%20Agreement).

**CITI Training**

***Collaborative Institutional Training Initiative (CITI):*** <https://www.research.miami.edu/about/admin-areas/raa/coi/citi/index.html>

The Collaborative Institutional Training Initiative (CITI Program) site offers web-based training in research compliance and ethics. CITI training is mandatory for all members of the research team.

**Login Instructions**:

1. Click on the “Login to CITI Program Using CaneID”
2. The CaneID Authentication Service page will appear
3. Enter your CaneID and Password and click on the “Sign In” button

***Note****: If this is the first time you have ever logged into CITI Program, a new CITI Program account is created for you.*

[Login to CITI Program Using CaneID](https://www.citiprogram.org/Shibboleth.sso/Login?target=https%3A%2F%2Fwww.citiprogram.org%2FSecure%2FWelcome.cfm&entityID=https%3A%2F%2Fcaneid.miami.edu%2Fidp%2Fshibboleth)

1. If you do not see the course you need, click on “Add or Update Learner Groups” in the University of Miami / Jackson Health System section of your CITI Program gradebook. This link is in the “My Learner Tools” section of the gradebook.
2. Complete the following course(s):
3. **Human Subjects Research Series (Question 3)**
   - ***Group 2: Social-Behavioral Researchers Basic Course (Question 8)***

**Motivational Interviewing**

CHWs will receive an introduction to Motivational Interviewing theory and practice specifically for HIV health care workers. The training series provides a didactic overview of the MI process and its practice, by the demonstration of MI, guided role playing of MI, evaluation of MI skills employed by self and colleagues, independent practice of MI, and coaching by MI instructors.

Below is the breakdown of the workshop series trainings and assignments:

| **Session** | **Session Topics** | **Homework** | **Skills Practice** |
| --- | --- | --- | --- |
| **1** | What is MI  MI in Healthcare/ HIV care  Spirit of MI (Compassion, Acceptance, Evocation & Collaboration/Partnership)  Process of MI (Engaging, focusing, evoking, planning) | Miller & Rollnick  pp.1-105 | Identify MI skills  (Observation & identification) |
| **2** | Focusing (Following, guiding, directing)  Tools of MI  Goal setting  Evoking (Ambivalence)  Demonstration of MI | Miller & Rollnick  pp. 106-180; pp. 181-210  List of personal & actual behavior changes due @ Session 3 | Practice Engaging  Practice Focusing  Practice Goal setting  Practice Evoking |
| **3** | Planning  Using MI to change behavior  Change talk/sustain talk/discord  Integrating MI in your visit | Miller & Rollnick  pp 211-290; pp 291-393  Standard practice /role play recording due @ Session 4 | Practice MI planning  Practice MI integration |
| **4** | Experiencing MI  MI fidelity- Self-awareness/coding  Training & Supervision | Miller & Rollnick  pp 368-369  Post-training patient consultation recording due 2-wks after Session 4 | Practice MI integration  Practice MI evaluation |

### Quality Assurance and Quality Control

The site will perform internal quality management of study conduct, data collection, documentation and completion. Quality control (QC) procedures will be completed by the Data Manager on a quarterly basis. Any missing data or data anomalies will be communicated to the Research Coordinator and CHWs for clarification/resolution. The site will provide direct access to all trial related sites, source data/documents, and reports for the purpose of monitoring and inspection by local and regulatory authorities.

### Data Handling and Record Keeping

#### Data Collection and Management Responsibilities

Data collection is the responsibility of the clinical trial staff at the site under the supervision of the Principal Investigator. The Principal Investigator is responsible for ensuring the accuracy, completeness, legibility, and timeliness of the data reported. All source documents should be completed in a neat, legible manner to ensure accurate interpretation of data.

#### Data safety Monitoring Plan

The investigators involved in the proposed project have completed the NIH required Data and Safety Monitoring Plan for Clinical Trials to ensure the safety of participants and the validity and integrity of the data. This study will be monitored internally by the UM Data and Safety Monitoring Committee every two months and all Adverse Events, Protocol Deviations, and Patient Enrollment will be reviewed and monitored. Additionally, the Clinical Research Operations & Regulatory Support (CRORS) Office will conduct monitoring of all study procedures including informed consent monitoring, review of source documentation and data, adverse events including significant adverse event reporting, monitoring of study drug storage and dispense logs, and review of protocol deviations. All Monitoring findings and recommendations will be reported directly to the PI as well as the Director of Clinical Research Services.

#### DSMB

The following section summarizes the Data Safety and Monitoring Committee at the University of Miami Miller School of Medicine (DSMC). DSMC is required for all types of clinical trials, including physiologic toxicity and dose-finding studies (phase I) efficacy studies (phase II), and effectiveness and comparative trials (phase III). At UM School of Medicine, the Protocol Review Monitoring Committee (PRMC) ensures that all trials include an appropriate DSMC. PRMC review occurs prior to IRB review to ensure the scientific merit of the proposed research as relates to the broader focus of UM research programs. For investigator-initiated interventional treatment studies, UM’s DSMC is adopted by investigators and implemented by a dedicated DSM Committee. Other investigator-initiated, interventional, non-treatment studies are reviewed on a case-by-case basis according to the PRMC’s risk assessment. The UM DSMC Plan relates to all trials conducted at the medical campus and places emphasis on data and safety monitoring of all investigator-initiated studies that do not have the provision of external monitoring. This plan was most recently updated in July 2017. The designated IRB

provides a final level of safety review for all protocols and continuation applications.

**DSMC Outcomes**

Following committee review of study activity, the DSMC will issue one of the following outcomes:

- Approved: The study can proceed.
- Contingently Approved: Clarifications or additional information is required; however, accrual activity may proceed.
- Suspension: Clarifications or additional information is required; however, trial accrual is suspended until issues and/or concerns noted are resolved and approved by full DSMC.
- Closure: The study is not approved, and closure is recommended.

**DSMC Composition**

DSMC membership consists of experienced investigators representing multiple specialties/disciplines. Sixty percent of current DSMC faculty members hold rank of associate or professor level and provide DSMC mentorship to assistant level faculty members.

#### Study Records Retention

Study documents will be retained for a minimum of 5 years after the completion of the study.

### Protocol Deviations

A protocol deviation is any noncompliance with the clinical trial protocol requirements. The noncompliance may be either on the part of the subject, the investigator, or the study site staff. As a result of deviations, corrective actions are to be developed by the site and implemented promptly.

These practices are consistent with ICH GCP:

- 4.5 Compliance with Protocol, sections 4.5.1, 4.5.2, and 4.5.3
- 5.1 Quality Assurance and Quality Control, section 5.1.1
- 5.20 Noncompliance, sections 5.20.1, and 5.20.2.

It is the responsibility of the Principal Investigator to use continuous vigilance to identify and report deviations within 10 working days of identification of the protocol deviation. Protocol deviations must be sent to the reviewing Institutional Review Board (IRB) per their policies. The Principal Investigator is responsible for knowing and adhering to the reviewing IRB requirements.

### Conflict of Interest Policy

The independence of this study from any actual or perceived influence, such as by the pharmaceutical industry, is critical. Therefore, any actual conflict of interest of persons who have a role in the design, conduct, analysis, publication, or any aspect of this trial will be disclosed and managed. Furthermore, persons who have a perceived conflict of interest will be required to have such conflicts managed in a way that is appropriate to their participation in the design and conduct of this trial.

### Dissemination Policy

(Any clinical trial as defined by the NIH conducted under the auspices of the University of Miami Institutional Review Board is registered in Clinical Trials.Gov no later than 21 calendar days after enrollment of the first participant. This trial will be registered in Clinicaltrials.gov. As required for all trials, the primary summary results of this study will be reported in ClinicalTrials.gov no later than one year after the primary completion date by Dr. Sonjia Kenya.

The informed consent documents for the clinical trial include a specific statement related to posting clinical trial information at ClinicalTrials.gov. The language included in the consent documents is as follows, “A description of this clinical trial will be available on http://www.ClinicalTrials.gov, as required by U.S. Law. This Web site will not include information that can identify you. At most, the Web site will include a summary of the results. You can search this Web site at any time.” This statement will be translated (using translation/back-translation procedures) to Spanish for our Spanish-language consents.

The University of Miami has an internal policy to ensure that clinical trials protocol registration occurs prior to the enrollment of the first participant. This applies when the University of Miami Principal Investigator has initiated, sponsored or been designated the Responsible Party for registering such Clinical Trials/Studies. Failure to comply with this policy may result in the following: inability to publish; civil monetary penalties levied against the University; suspension of protocol approval from the IRB; loss of additional or continued funding from federal agencies and other entities.
